# Establishing an RNA fusions panel in soft tissue sarcoma with clinical validation

**DOI:** 10.1038/s41598-023-29511-1

**Published:** 2023-03-16

**Authors:** Xiaoqiang Huang, Guibin Li, Linghua Li, Jian Wang, Jianru Shen, Yao Chen, Wuzhong Yu, Ailin Chen, Tao Wu, Ji Ma, Bao Ling, Liang He, Xudan Chen

**Affiliations:** 1 Guangzhou KingMed Center for Clinical Laboratory Co. Ltd., Guangzhou, China; 2 Guangzhou KingMed Diagnostics Group Co. Ltd., Guangzhou, China; 3 Guangzhou KingMed Transformative Medicine Institute Co. Ltd., Guangzhou, China; 4 Guangzhou Eighth People’s Hospital, Guangzhou Medical University, Guangzhou, China; 5 Shenzhen KingMed Medical Laboratory, Shenzhen, China

**Keywords:** Biological techniques, Cancer, Genetics, Molecular biology

## Abstract

The diagnosis and classification of soft tissue sarcomas (STS) remain challenging because of the rarity and overlapping morphologic manifestations of diverse STS subtypes. Characteristic gene fusions are commonly detected in STS and represent useful diagnostic markers. This study established and validated a custom-designed RNA sequencing panel that identified 64 gene fusions in STS. The analytical performance validation yielded excellent accuracy, with 100% (95% CI, 94.40%-100%) sensitivity and 93.33% (95% CI, 68.05%-99.83%) specificity. Clinical performances were further confirmed with 145 clinical formalin-fixed and paraffin-embedded (FFPE) samples from STS patients. Fusions were detected in 40% of samples (58/145). The common fusions *SS18-SSX* family, *EWSR1*-related fusions, *COL1A1-PDGFB*, *FOXO1*-associated fusions, and *FUS*-associated fusions were identified in corresponding STS subtypes. The RNA panel detected specific fusions in several cases where no conclusive diagnosis can be made based on the morphology and immunohistochemistry results. Data collected in this study demonstrate that the RNA fusions panel can better classify STS subtypes and serve as a good supplement for histopathology, exhibiting a great potential for the STS precise diagnosis.

## Introduction

Soft tissue sarcomas (STS), an extremely heterogeneous form of rare tumors that affect different tissue structures, are most commonly found in the extremities, followed by the trunk, retroperitoneum and abdomen, head, and neck^1–3^. Depending on morphology, STS contains more than 70 histologic subtypes, which signifies a particular challenge for diagnostic histopathology^4–8^.

Advances in molecular genetic studies have provided significant insights into the molecular mechanisms of STS^9–11^. One of the most important mechanisms that drive sarcoma tumorigenesis lies in the transcriptional dysregulation caused by aberrant fusion proteins resulting from genomic rearrangements^12,13^. Many sarcomas subtypes harbor characteristic gene fusions, such as the myogenic transcription factor genes *PAX3* and *PAX7* joining with *FOXO1* in alveolar rhabdomyosarcomas (ARMS)^14^. It’s reported that fusion-associated sarcomas comprise an estimated 30% of all sarcomas, and NCCN guidelines recommend 44 specific fusion genes as diagnostic markers^6,11^. Here, the detection of gene fusions has greatly facilitated STS diagnosis and subclassification in cases where histopathology review has been indeterminate^15^.

Gene fusions are usually identified at the DNA level by identifying the breakpoints. But, DNA-based detection assays have limitations. First of all, as the causal genomic breakpoints are unpredictable, DNA-based fusion detections need to analyze a wide range of introns. The efficiency of gene fusions detected by DNA sequencing remained low. It was reported that fusions were only found at 10.7% (7/65), 11.6% (68/584), and 17.8% (18/102) in STS patients by a DNA sequencing panel^15–17^. Second, a fraction of uncommon fusions proved to generate no aberrant transcripts or proteins^18,19^. Further RNA sequencing revealed that 12.8% of uncommon fusions identified by DNA-based NGS were nonproductive^18^.

Upon integration with RNA data, we can determine whether the effect of a given fusion is predominantly repressive or activating. The main objective of our study is to set up a highly efficient RNA fusion panel and to evaluate the potential of molecular classification in STS. Analytical performance evaluations based on the reference materials were presented. Clinical validation of the RNA fusion panel was also discussed.

## Materials and methods

### Samples

The study was conducted by the Declaration of Helsinki and was approved by the Ethics Committee of Guangzhou KingMed Diagnostics Group. The Ethics Committee concluded that no informed consent was required because data were anonymized appropriately in the retrospective study (IRB-2022012). 145 FFPE tissue specimens from Chinese STS patients were analyzed in this study, all of whom were diagnosed in multiple hospitals from April 2019 to Dec. 2021 according to the clinical guidelines^20^.

### Panel design

A total of 64 gene fusions were chosen as clinically significant targets in STS isoforms, based on the COSMIC database, NCCN guidelines, and public research^21–24^.

The fusion genes targeted in this panel are listed in Table S1. Transcript sequence data were obtained from the COSMIC database and public literature. Gene‐specific primers were designed to span the exon-exon junctions of the fusion transcripts. Primers covered both fusion genes and partner genes.

### Library preparation and fusion detection

Ion AmpliSeq™ RNA workflow was deployed for the detection of fusion transcripts in the study. Total RNA was extracted using RecoverAll™ Total Nucleic Acid Isolation Kit (Invitrogen, USA) from 5 µm FFPE sections. 10–50 ng ng of total RNA was reverse-transcribed into cDNA. Fusion-specific primers panel were used to amplify targets with the Ion AmpliSeqTM RNA Library Kit. Unincorporated primers were digested away, and sequencing adapters ligated onto libraries. RNA libraries were sequenced on the Ion PGM™ system and Ion 318™ chip. After sequencing, Ion Reporter™ v.5.6 was used for the analysis of the fusion transcripts. Preliminary data were analyzed to give the number of counts per amplicon in the RNA fusion Panel. The cutoff was fusion read count per 1000, 000 (CP1000K) > 2175 and support reads > 87, with the total mapped reads ≥ 40,000.

A false-negative result could occur if the RNA was degraded seriously. It is important to have an indicator in place for confirming sample RNA quality. The RNA workflow included primer sets for 10 endogenous reference genes to determine if the quality of the results could be affected by RNA quality. These endogenous genes were moderately but widely expressed across different human tissues. The amplicon length of the internal reference genes was larger than the potential fusion variant amplicon. If ≧ 6 internal reference genes could be detected successfully, the sample passed the quality control.

### Analytical performance validation

We used cell lines from the College of American Pathologists (CAP), FFPE samples from clinical STS patients, and synthetic fusion gene fragments as the reference sample set for accuracy validation (Table S2). 17 well-characterized cell lines with known gene fusions from the CAP SARComa or RNA sequencing proficiency test (PT) were also included. Besides, 48 fusion constructs were synthesized by GENEWIZ Inc. (Suzhou, China), with the junction sequence of fusions as a template. 14 FFPE sections from STS patients were included in this part, on which fluorescence in situ hybridization (FISH) assays had been performed using break-apart probes to detect one of the chromosomal rearrangements: *EWSR1*, *FOXO1*, *FUS*, or *SS18* translocation. FISH signals were assessed under an Olympus BX51TRF microscope (Olympus, Japan). Signals were considered to be split when the distance between red and green signals ≥ 3 signal diameters. Cells without the rearrangement had two sets of red and green fusion signals indicating intact chromosomes. For each case, a minimum of 200 tumor nuclei were evaluated by two independent operators. A positive result was reported when ≥ 10% of the tumor nuclei had break-apart signals.

The reference material was analyzed using RNA‐NGS, then assessed if they aligned with the expected results.

### Fusion confirmation by Sanger sequencing

Sanger sequencing, an orthogonal method, was applied to confirm the gene fusion when an inconsistency occurred between our panel and the reference method. Gene-specific primers were designed according to the fusion sequence determined by the RNA fusion sequencing (Table S3). RNA was transcribed into cDNA and then performed PCR reaction using the AmpliTaq Gold™ 360 Mater Mix (Applied Biosystems, Cat# 4398876) following the manual. DNA sequencing was performed by gold-standard Sanger sequencing, using Applied Biosystems 3500 Genetic Analyzer in coordination with the manual. Sequence data were analyzed using Sequencher (V.5.0; Gene Codes Corporation, Ann Arbor, MI, USA), and the consensus sequence was aligned to human genomic and transcript databases using NCBI BLASTn 2.80.

### Clinical validation

145 STS samples were collected and used for validating the RNA panel, which were from surgically resected tissue, routinely processed and embedded in paraffin, most from thighs and hands (Table S4).

The Department of Pathology at KingMed Diagnostics performed a histopathological diagnosis of the STS tissue following the World Health Organization (WHO) standards. Hematoxylin and eosin-stained slides of STS specimens were reviewed by an experienced pathologist. Immunohistochemistry (IHC) assays were conducted to facilitate the histopathological classification.

The main steps of IHC include antigen retrieval, primary antibody binding, secondary antibody binding and 3,3' Diaminobenzidine (DAB) staining. The IHC staining for protein biomarkers was semi-quantitatively scored as “−” (negative, no, or < 5% positive cells), “+” (6–50% positive cells), and “++” (< 50% positive cells, considered as strongly positive). An expert pathology consultation will be incorporated into the histopathologic evaluation process when it encounters complicated specimens.

### Ethics approval

All procedures were performed according to the Declaration of Helsinki and were approved by the Ethics Committee of Guangzhou KingMed Diagnostics center (IRB number 2022012). The Ethics Committee concluded that no informed consent was required because the data were anonymized appropriately.

## Results

### Accuracy validation of the RNA fusion panel

The method proposed in the study was validated using 48 synthetic DNA constructs and 31 samples with known fusions (17 PT samples from CAP, 14 samples tested by FISH assays), and the information for the 79 samples was referenced in Supplemental Table S2.

The accuracy (overall percent agreement) was reported at 98.73% (95% CI 93.15–99.97%) compared with FISH and reference material. The sensitivity and specificity (positive and negative percent agreements) were 100% (95% CI 94.40–100%) (64/64) and 93.33% (95% CI 68.05–99.83%), respectively (Table 1). The adjusted c^2^ value is 0, indicating that the RNA NGS was highly consistent with FISH and reference material.

**Table 1:** Chi-square test of quadruple tabular form.

STS RNA fusion	Reference material		Subtotal
	Positive	Negative	
Positive	64	1	65
Negative	0	14	14
Subtotal	64	15	79

**Figure 1:**
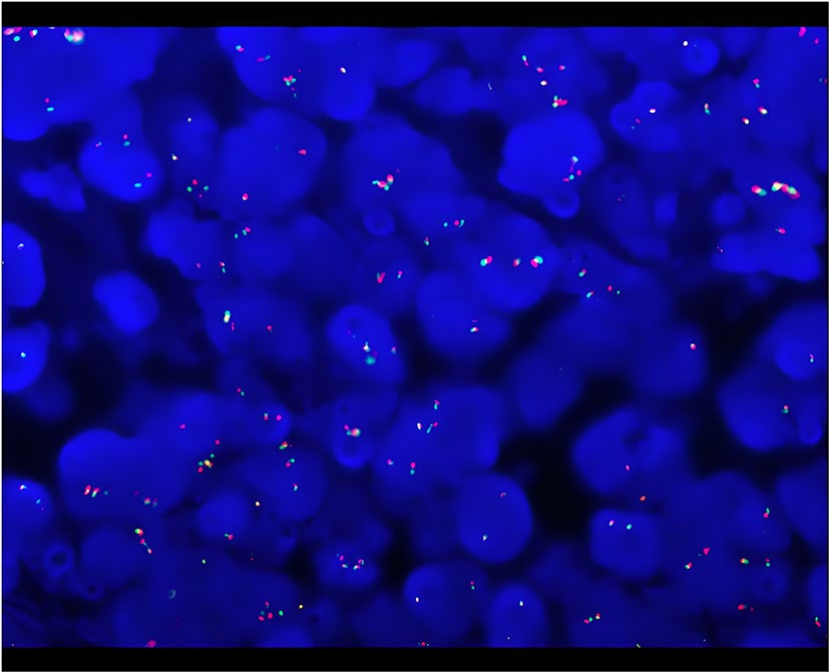
*EWSR1* break-apart FISH analysis showed split signals in several cells. Red probes are designed to hybridize to the 5' region of *EWSR1* and green probes to the 3' one.

One sample which reported negative in the FISH analysis was detected as positive by our method. FISH tests did not detect *EWSR1* or *FOXO1A* separation and rearrangement. However, our RNA sequencing uncovered an *EWSR1*-*WT1* fusion in this sample, with 25,875 fusion read counts (87,307 total mapped reads). For this sample, Sanger sequencing was performed, and BLAST results showed that the first half of the amplicon sequence shared 97.3% identity with Homo sapiens EWS RNA binding protein 1 (*EWSR1*), transcript variant 1, mRNA, and the second half of the amplicon sequence shared 100% identity with Homo sapiens *WT1* transcription factor, transcript variant D, mRNA. The evidence demonstrated that the FISH test result of this case was false-negative. FISH images of this sample were rechecked and several break-apart signals (Fig. 1) were found. But the section quality of this sample was of poor quality so cells were prone to accumulate and stack, with blurred cell boundaries which were not easy to count individually.

This sample was obtained from a patient who presented with a multinodular pelvic peritoneum mass with multiple involvements of adrenal glands, liver, and lymph nodes. The pathologist considered it to be a small blue round cell tumor with proliferation of interstitial fibrous tissue from the histologic morphology. The presence of an *EWSR1*-*WT1* gene fusion is characteristic of a desmoplastic small round cell tumor, a morphologically diverse malignant stromal tumor^25^.

This sample suggests that false negatives may be generated by the low sensitivity of the FISH assay, which is of great concern. Our RNA panel could overcome the disadvantage of FISH testing.

### Evaluation of clinical diagnostic efficacy

This study aims to investigate the efficiency of the RNA fusion panel in STS diagnostics as molecular classification. FFPE from 145 Chinese STS patients were analyzed. Regarding histological subtypes, the study contained diverse STS subtypes as Table S5.

**Figure 2:**
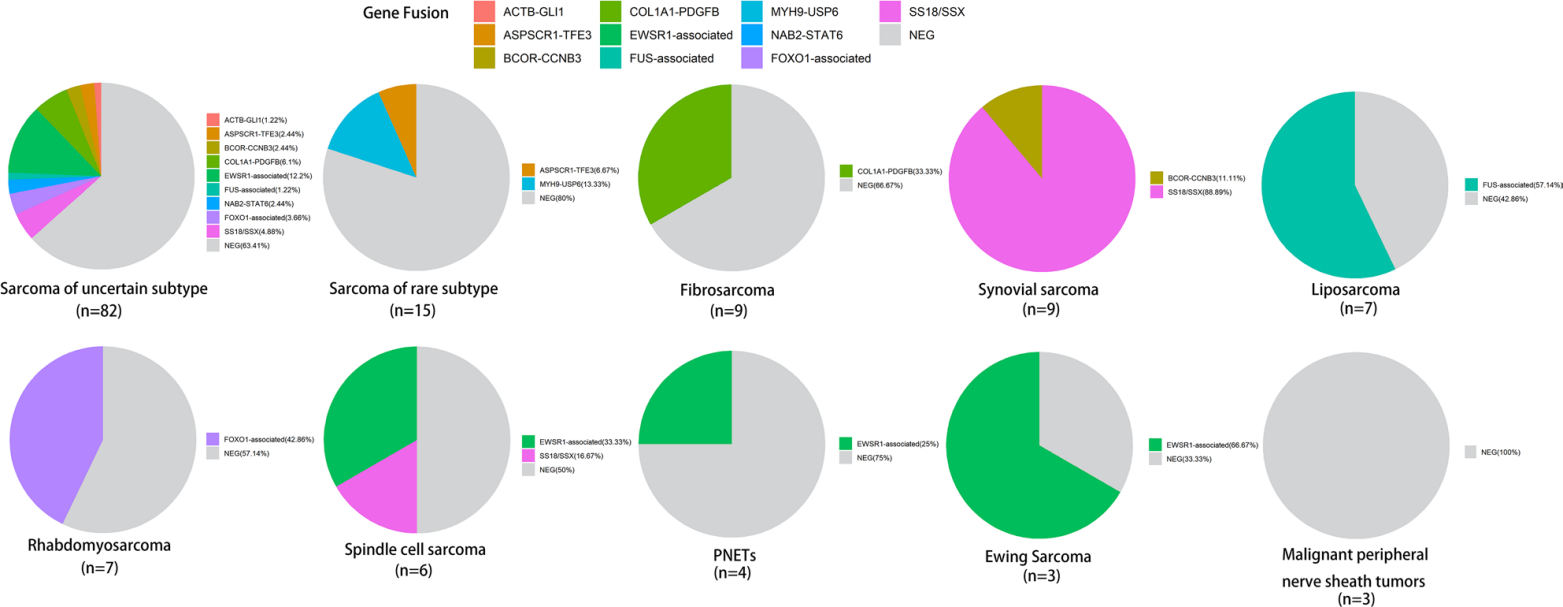
Distribution and characteristics of gene fusions in 145 Chinese STS patients. The fusion types were shown, including *COL1A1-PDGFB* fusion, *EWSR1* fusions, *FUS* fusions, *FOXO1* fusions, and *SS18-SSX* fusions, as labeled above. The histological subtypes for each patient are shown at the bottom of the chart. Additional information about data in this figure is provided in Tables S4. NEG denotes negative, meaning no fusions were detected. Patients with unknown histology indicated that a definitive pathological subtype could not be determined based on pathological examinations, like patient No.28 below.

RNA from the tissue biopsies was analyzed by the targeted RNA fusion panel. Fusions were observed in 40% (58/145) of STS patients (Table S4). Fusion genes were composed of (i) *SS18-SSX* family (including S*S18-SSX1*, *SS18-SSX2,* and *SS18-SSX4*, n = 13), (ii) *EWSR1*-associated fusions (n = 15), (iii) *COL1A1-PDGFB* (n = 8), (iv) *FOXO1*-associated fusions (n = 6), and (v) *FUS*-associated fusions (n = 5), (vi) *BCOR-CCNB3* (n = 3), (vii) *ASPSCR1-TFE3* (n = 3), *MYH9-USP6* (n = 2), *NAB2-STAT6* (n = 2) and *ACTB-GLI1* (n = 1), respectively (Fig. 2). The common fusions were identified in corresponding STS subtypes, e.g., *FOXO1*-associated fusions in rhabdomyosarcoma from the head or neck.

**Figure 3:**
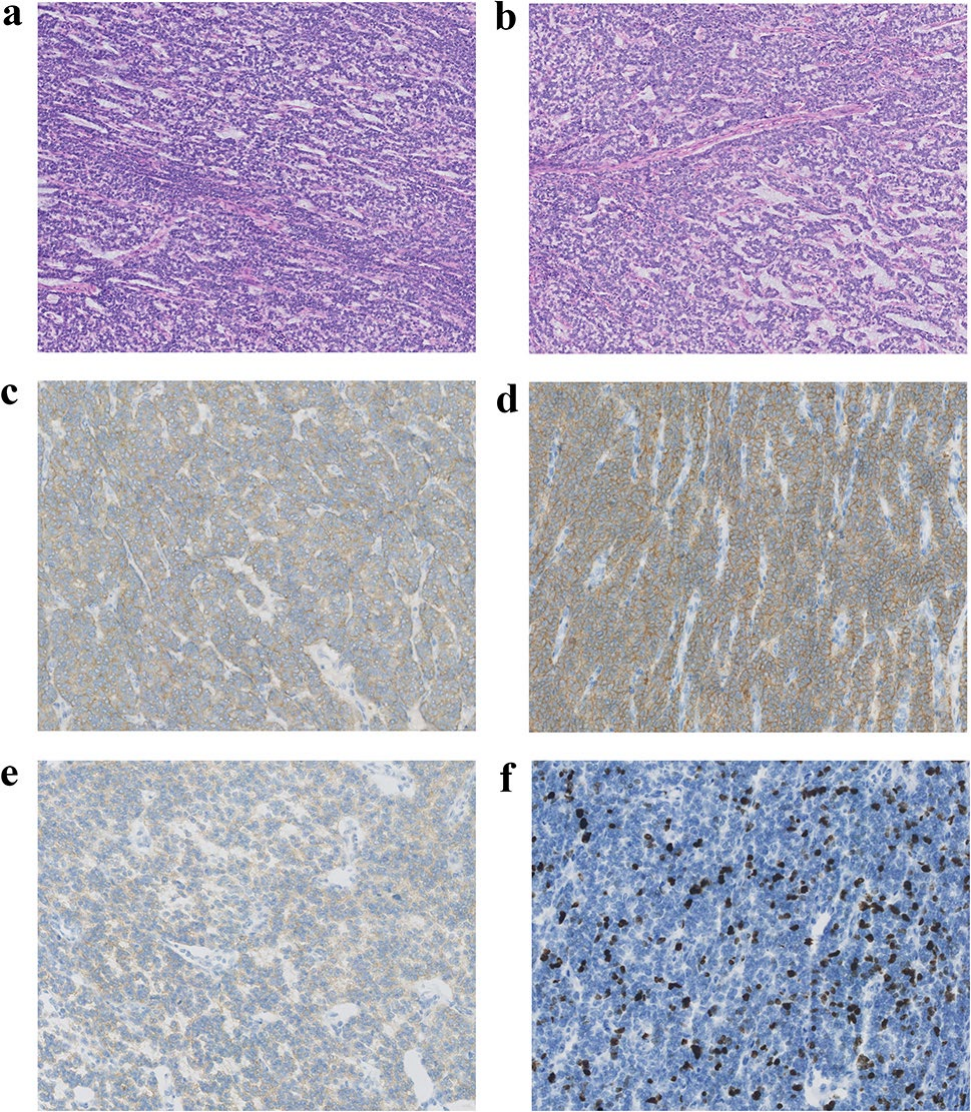
Hematoxylin and eosin staining (HE) and IHC of No.28 sample. Histological images of tumor from gastric wall: (**a**) In 100 × magnification, monomorphic cells with round to oval nuclei were observed. (**b**) In 100 × magnification, neoplastic cells formed acinar-like structures with the myxoid background. Tumor samples were further characterized by immunohistochemistry for the expression of specific markers. (**c**) In 200 × magnification, CD10 expression was observed in the neoplastic cells. (**d**) In 200 × magnification, CD56 expression in the neoplastic cells. (**e**) In 200 × magnification, CD117 has weak positivity in the neoplastic cells. (**f**) In 200 × magnification, elevated Ki-67 proliferative index in the neoplastic cells.

Among 145 samples, patient No.28 was of interest as conventional IHC staining identified tumor cells positive for CD56, CD10, and CD117, with a high Ki67 index in some areas (~ 50%) (Fig. 3). The tumor cells were negative for AE1/3, Cam5.2, CKIT, DOG1, CD99, SOX10, S100, HMB45, MelanA, Syn, CgA, Trypsin, Desmin, SMA, Caldesmon, collagen type 4, Laminin, Inhibin, Calretinin, STAT6, CD34, ERG, WT1, ER, PR, and SALL4. Histologically, the tumor from the gastric wall was cellular, well-demarcated, and composed of fairly monomorphic cells with round to oval nuclei and areas of clear cell change vs retraction artifact. In some areas, the cells appeared to be forming acinar-like structures. Some areas appear a bit myxoid. There is a somewhat prominent background capillary network.

Diagnoses that were essentially excluded based on the IHC results include carcinoma, melanoma, neuroendocrine neoplasm, smooth muscle tumor, GIST, glomus tumor, adrenal cortical carcinoma, solitary fibrous tumor, and vascular neoplasm, endometrial stromal sarcoma, germ cell tumor, and solid pseudopapillary tumor.

It's considered an unusual mesenchymal neoplasm. This case was sent for consultation at the University of Pittsburgh Medical Center before being sent to our laboratory. Pathologists believed it may be round cell/myxoid liposarcoma (in some areas the vascular pattern could be construed as “chicken-wire” vasculature, though it was S100-negative) or epithelioid synovial sarcoma. The morphology was not typical for gastroblastoma and was negative for CD99, ERG, and WT1, which were against CIC/DUX sarcoma. But no diagnosis can be made based on the inconclusive morphology and immunohistochemistry results. In this case, our laboratory identified this patient with an *ACTB-GLI1* fusion. A literature search revealed that *ACTB-GLI1* appears in part of perithelial cell tumors^26,27^.

## Discussion

In this study, we reveal a fusion detection method based on RNA sequencing, which outperforms FISH in the detection of gene fusions present in STS.

The heterogeneity of STS concerning molecular genesis, histology, clinical characteristics, and treatment response makes managing these rare yet diverse neoplasms, particularly challenging^28–30^. As a result of the incorrect initial diagnosis, inappropriate medical management has been reported in sarcoma patients^4,31,32^. Therefore, histopathologic reviews for soft tissue sarcomas are still recommended in recent years^32,33^. But diagnostic difficulties still exist. First of all, experienced pathologists, especially anatomical pathologists, are still unavailable in many medical institutes in China. According to the Statistical Bulletin for China’s Health Care Development in 2019 published on the official website of the National Health Commission, domestic hospitals had 6.867 million beds, and only 17,000 pathologists were registered, 70% of which were concentrated in tertiary hospitals^34^.

Based on 1–2 pathologists per 100 beds, the shortage is as high as 50,000–100,000 pathologists.

Second, it is clear that various differentiations can co-exist within the tissue of a single tumor, so inter-observer differences are inevitable when making a pathological diagnosis based on only morphologic features^35^. In the study, the pathological subtypes were unclear in 56.5% of the STS cohort. This reflected that half of the pathologists in China do not have sufficient experience in diagnosing STS.

In practice, histopathology examination requires a high level of expertise and knowledge. Pathologists have a long training cycle, at least 3–5 years, and need to be familiar with the morphology of 50,000–100,000 pathology specimens. For some rare and difficult tumors (about 20–30%), pathologists may be trained for more than 5 years to make an accurate diagnosis.

Even experienced pathologists of sarcoma could make mistakes in the diagnosis of this disease. Research showed about 30% of initial clinical diagnoses of newly described STS were changed according to identified fusions^36,37^.

Fusion detection by NGS could be used to differentiate the histologic subtypes of STS. In this study, RNA-based gene fusions were detected in 40% of STS patients, which was significantly more than that identified by DNA-based sequencing. STS patients' most frequent fusion genes included the *SS18-SSX* family, *EWSR1*-associated fusions, *COL1A1*-*PDGFB*, *FOXO1*-associated fusions, etc. And we also successfully detected some rare fusion genes, like, *MYH9-USP6* and *ACTB-GLI1*. Through the detection of translocation t (7;12) (p22; q13), which induces the fusion of the *ACTB* and *GLI* gene, a newly soft tissue tumor is defined as "Pericytoma with t (7;12)"^38^.

Pericytoma is a rare tumor, which is even rarer if it occurs in the stomach. Our study analyzed a stomach tumor sample resected from a 48-year-old female, located at the serosa of the lesser curvature. According to the pathology report, no specific diagnosis can be made based on the histopathological and IHC results, but it favored sarcoma. But part of the tumor cells was CD117 immunostaining weakly positive, and CD10 positive, another pathologist thought gastrointestinal stromal tumors should be considered. In this case, the inconclusive morphology features and controversial immunohistochemistry results confused the clinician. And in our laboratory, *ACTB*-*GLI1* fusion was detected in the sample, which offered definitive evidence for diagnosing this tumor as pericytoma with t (7;12) of the stomach. The data reflect the high impact of molecular markers on STS classification, and the RNA gene fusion panel should play a more important role in characterizing challenging cases.

## Conclusion

In summary, an efficient RNA-targeted panel for the identification of gene fusions in soft tissue sarcomas was set up in the study.

We applied this panel to the clinical setting and found that the RNA fusion panel represents a substantial improvement in sensitivity and accuracy compared to FISH in detecting gene fusions and may reveal unexpected rearrangements in rare STS subtypes.

There are some limitations to our STS RNA fusion panel.

Given that certain gene fusions may be shared by many different tumor types and 60%, or more of sarcomas are not driven by characteristic gene fusions, this, and similar molecular techniques are not a substitute for histologic morphological examination with immunohistochemistry. Alternative solutions need to be found for the lack of experienced pathologists, such as the establishment of reference pathologists in each region coupled with telemedicine.

In addition, this panel included only known fusions. The fusion panel will be continuously upgraded by incorporating new fusion genes in the future.

Despite this, we have shown positive and promising results. Targeted RNA sequencing is proved to be a powerful tool for a comprehensive review of gene fusions in STS.

## Supplementary Information

Supplementary Tables.

Supplementary Table S2.

Supplementary Table S4.

## Data Availability

All data generated or analyzed during this study are included in this published article and its supplementary information files.

## References

[1] Zhou M, Wang H, Zeng X, Yin P, Zhu J, Chen W (2019). Mortality, morbidity, and risk factors in China and its provinces, 1990–2017: A systematic analysis for the Global Burden of Disease Study 2017. Lancet.

[2] Coindre JM, Terrier P, Bui NB, Bonichon F, Collin F, Le Doussal V (1996). Prognostic factors in adult patients with locally controlled soft tissue sarcoma. A study of 546 patients from the French Federation of Cancer Centers Sarcoma Group. J. Clin. Oncol..

[3] Cormier JN, Pollock RE (2004). Soft tissue sarcomas. CA.

[4] Jo VY, Fletcher CD (2014). WHO classification of soft tissue tumours: An update based on the 2013 (4th edition). Pathology.

[5] Kallen ME, Hornick JL (2021). The 2020 WHO Classification: What's new in soft tissue tumor pathology?. Am. J. Surg. Pathol..

[6] von Mehren M, Kane JM, Bui MM, Choy E, Connelly M, Dry S (2020). NCCN guidelines insights: Soft tissue sarcoma, Version 1.2021. J. Natl. Compr. Cancer Netw..

[7] Burningham Z, Hashibe M, Spector L, Schiffman JD (2012). The epidemiology of sarcoma. Clin. Sarcoma Res..

[8] Kelleher FC, Viterbo A (2013). Histologic and genetic advances in refining the diagnosis of "undifferentiated pleomorphic sarcoma". Cancers.

[9] Jour G, Scarborough JD, Jones RL, Loggers E, Pollack SM, Pritchard CC (2014). Molecular profiling of soft tissue sarcomas using next-generation sequencing: A pilot study toward precision therapeutics. Hum. Pathol..

[10] Movva S, Wen W, Chen W, Millis SZ, Gatalica Z, Reddy S (2015). Multi-platform profiling of over 2000 sarcomas: Identification of biomarkers and novel therapeutic targets. Oncotarget.

[11] Xu LB, Zhao ZG, Xu SF, Zhang XX, Liu T, Jing CY (2020). The landscape of gene mutations and clinical significance of tumor mutation burden in patients with soft tissue sarcoma who underwent surgical resection and received conventional adjuvant therapy. Int. J. Biol. Markers.

[12] Mossé YP, Voss SD, Lim MS, Rolland D, Minard CG, Fox E (2017). Targeting ALK with crizotinib in pediatric anaplastic large cell lymphoma and inflammatory myofibroblastic tumor: A children's oncology group study. J. Clin. Oncol..

[13] Yamazaki F, Nakatani F, Asano N, Wakai S, Sekimizu M, Mitani S (2019). Novel NTRK3 fusions in fibrosarcomas of adults. Am. J. Surg. Pathol..

[14] Mercado GE, Barr FG (2007). Fusions involving PAX and FOX genes in the molecular pathogenesis of alveolar rhabdomyosarcoma: Recent advances. Curr. Mol. Med..

[15] Jin G, Wang C, Jia D, Qian W, Yin C, Wang D (2021). Next generation sequencing reveals pathogenic and actionable genetic alterations of soft tissue sarcoma in Chinese patients: A single center experience. Technol. Cancer Res. Treat..

[16] Groisberg R, Hong DS, Holla V, Janku F, Piha-Paul S, Ravi V (2017). Clinical genomic profiling to identify actionable alterations for investigational therapies in patients with diverse sarcomas. Oncotarget.

[17] Lucchesi C, Khalifa E, Laizet Y, Soubeyran I, Mathoulin-Pelissier S, Chomienne C (2018). Targetable alterations in adult patients with soft-tissue sarcomas: Insights for personalized therapy. JAMA Oncol..

[18] Li W, Guo L, Liu Y, Dong L, Yang L, Chen L (2021). Potential unreliability of uncommon ALK, ROS1, and RET genomic breakpoints in predicting the efficacy of targeted therapy in NSCLC. J. Thorac. Oncol..

[19] Li W, Liu Y, Li W, Chen L, Ying J (2020). Intergenic breakpoints identified by DNA sequencing confound targetable kinase fusion detection in NSCLC. J. Thorac. Oncol..

[20] von Mehren M, Randall RL, Benjamin RS, Boles S, Bui MM, Ganjoo KN (2018). Soft tissue sarcoma, Version 2.2018, NCCN clinical practice guidelines in oncology. J. Natl. Compr. Cancer Netw..

[21] Errani C, Zhang L, Sung YS, Hajdu M, Singer S, Maki RG (2011). A novel WWTR1-CAMTA1 gene fusion is a consistent abnormality in epithelioid hemangioendothelioma of different anatomic sites. Genes Chromosom. Cancer.

[22] Erickson-Johnson MR, Chou MM, Evers BR, Roth CW, Seys AR, Jin L (2011). Nodular fasciitis: A novel model of transient neoplasia induced by MYH9-USP6 gene fusion. Lab. Investig..

[23] Pierron G, Tirode F, Lucchesi C, Reynaud S, Ballet S, Cohen-Gogo S (2012). A new subtype of bone sarcoma defined by BCOR-CCNB3 gene fusion. Nat. Genet..

[24] Walther C, Tayebwa J, Lilljebjörn H, Magnusson L, Nilsson J, von Steyern FV (2014). A novel SERPINE1-FOSB fusion gene results in transcriptional up-regulation of FOSB in pseudomyogenic haemangioendothelioma. J. Pathol..

[25] Lee JC, Villanueva-Meyer J, Ferris SP, Cham EM, Zucker J, Cooney T (2020). Clinicopathologic and molecular features of intracranial desmoplastic small round cell tumors. Brain Pathol..

[26] Bridge JA, Sanders K, Huang D, Nelson M, Neff JR, Muirhead D (2012). Pericytoma with t(7;12) and ACTB-GLI1 fusion arising in bone. Hum. Pathol..

[27] Dahlén A, Fletcher CDM, Mertens F, Fletcher JA, Perez-Atayde AR, Hicks MJ (2004). Activation of the GLI oncogene through fusion with the beta-actin gene (ACTB) in a group of distinctive pericytic neoplasms: Pericytoma with t(7;12). Am. J. Pathol..

[28] In GK, Hu JS, Tseng WW (2017). Treatment of advanced, metastatic soft tissue sarcoma: Latest evidence and clinical considerations. Ther. Adv. Med. Oncol..

[29] Judson I, Verweij J, Gelderblom H, Hartmann JT, Schöffski P, Blay JY (2014). Doxorubicin alone versus intensified doxorubicin plus ifosfamide for first-line treatment of advanced or metastatic soft-tissue sarcoma: A randomised controlled phase 3 trial. Lancet Oncol..

[30] Frezza AM, Stacchiotti S, Gronchi A (2017). Systemic treatment in advanced soft tissue sarcoma: What is standard, what is new. BMC Med..

[31] Arbiser ZK, Folpe AL, Weiss SW (2001). Consultative (expert) second opinions in soft tissue pathology. Analysis of problem-prone diagnostic situations. Am. J. Clin. Pathol..

[32] Lurkin A, Ducimetière F, Vince DR, Decouvelaere A-V, Cellier D, Gilly FN (2010). Epidemiological evaluation of concordance between initial diagnosis and central pathology review in a comprehensive and prospective series of sarcoma patients in the Rhone-Alpes region. BMC Cancer.

[33] Ray-Coquard I, Montesco MC, Coindre JM, Dei Tos AP, Lurkin A, Ranchère-Vince D (2012). Sarcoma: Concordance between initial diagnosis and centralized expert review in a population-based study within three European regions. Ann. Oncol..

[34] Commission NH. *Statistical Bulletin for China’s Health Care Development in 2019* (2020).

[35] Perry A, Burton SS, Fuller GN, Robinson CA, Palmer CA, Resch L (2010). Oligodendroglial neoplasms with ganglioglioma-like maturation: A diagnostic pitfall. Acta Neuropathol..

[36] Zhao G, Xie L, Guo W, Xi Y, Cui Y, Yu S (2020). The impact of next generation sequencing on sarcoma diagnosis. J. Clin. Oncol..

[37] El-Deiry WS, Goldberg RM, Lenz HJ, Shields AF, Gibney GT, Tan AR (2019). The current state of molecular testing in the treatment of patients with solid tumors, 2019. CA.

[38] Dahlén A, Mertens F, Mandahl N, Panagopoulos I (2004). Molecular genetic characterization of the genomic ACTB-GLI fusion in pericytoma with t(7;12). Biochem. Biophys. Res. Commun..

