# Supplementary material for: Establishing an RNA fusions panel in soft tissue sarcoma with clinical validation
Source: Sci Rep. 2023 Mar 16;13:4403. doi: 10.1038/s41598-023-29511-1 (PMC10020547; doi:10.1038/s41598-023-29511-1)
Supplement: Supplementary file 1 — Supplementary Tables. [file 41598_2023_29511_MOESM1_ESM.docx]

Table S1. Gene fusions targeted in the RNA panel

| *ACTB-GLI1* | *COL1A1-USP6* | *EWSR1-FEV* | *FUS-CREB3L2* | *OMD-USP6* | *TPM3-NTRK1* |
| --- | --- | --- | --- | --- | --- |
| *ASPSCR1-TFE3* | *COL1A2-PLAG1* | *EWSR1-FLI1* | *FUS-DDIT3* | *PAX3-FOXO1* | *TPM4-ALK* |
| *ATIC-ALK* | *EPC1-PHF1* | *EWSR1-NFATC2* | *FUS-ERG* | *PAX3-NCOA1* | *TPR-NTRK1* |
| *BCOR-CCNB3* | *ETV6-NTRK3* | *EWSR1-NR4A3* | *HAS2-PLAG1* | *PAX3-NCOA2* | *WWTR1-CAMTA1* |
| *CARS-ALK* | *EWSR1-ATF1* | *EWSR1-PBX1* | *HEY1-NCOA2* | *PAX7-FOXO1* | *ZC3H7B-BCOR* |
| *CDH11-USP6* | *EWSR1-CREB1* | *EWSR1-POU5F1* | *HMGA2-LPP* | *RANBP2-ALK* | *EWSR1-PATZ1* |
| *CIC-DUX4* | *EWSR1-DDIT3* | *EWSR1-SMARCA5* | *LMNA-NTRK1* | *SERPINE1-FOSB* | *SFPQ-TFE3* |
| *CIC-FOXO4* | *EWSR1-ERG* | *EWSR1-SP3* | *MEAF6-PHF1* | *TAF15-NR4A3* | *TCF12-NR4A3* |
| *CLTC-ALK* | *EWSR1-ETV1* | *EWSR1-ZNF444* | *MYH9-USP6* | *TFG-NR4A3* | *EWSR1-WT1* |
| *COL1A1-PDGFB* | *EWSR1-ETV4* | *FUS-ATF1* | *NAB2-STAT6* | *TPM3-ALK* | *THRAP3-USP6* |
| *SS18-SSX1* | *SS18-SSX2* | *SS18-SSX4* | *CNBP-USP6* |  |  |

Table S3. Primer sequences for fusion confirmation

| **Gene fusion** | **Forward primer** | **Reverse primer** |
| --- | --- | --- |
| EWSR1-WT1 | 5’- TGTAAAACGACGGCCAGTTATAGCCAACAGAGCAGCAG-3’ ^¶^ | 5’-  CAGGAAACAGCTATGACCTGGTGTCTTTTGAGCTGGTC-3’ ^¶^ |

^¶^ M13 sequences are underlined.

Table S5. The histological subtype of the Chinese STS patient’s cohort (n = 145).

| **Histological subtype** | **NO.** | **Percent (%)** |
| --- | --- | --- |
| Sarcoma of uncertain subtypes | 82 | 56.55% |
| Fibrosarcoma | 9 | 6.21% |
| Synovial sarcoma | 9 | 6.21% |
| Liposarcoma | 7 | 4.83% |
| Rhabdomyosarcoma | 7 | 4.83% |
| Spindle cell sarcoma | 6 | 4.14% |
| Primitive neuroectodermal tumors (PNETs) | 4 | 2.76% |
| Ewing Sarcoma | 3 | 2.07% |
| Malignant peripheral nerve sheath tumors | 3 | 2.07% |
| Melanoma or sarcoma | 2 | 1.38% |
| Histiocytoma | 2 | 1.38% |
| Myofibroblastic sarcoma | 2 | 1.38% |
| Myxoid fibrosarcoma or nodular fasciitis | 2 | 1.38% |
| Leiomyosarcoma | 1 | 0.69% |
| Schwannoma | 1 | 0.69% |
| Clear cell sarcoma | 1 | 0.69% |
| Osteosarcoma | 1 | 0.69% |
| Epithelioid sarcoma | 1 | 0.69% |
| Undifferentiated pleomorphic sarcoma | 1 | 0.69% |
| Alveolar soft tissue sarcoma | 1 | 0.69% |
